# An Exploration of Differences Between Deliberate Self-Harm with and without Suicidal Intent Amongst a Clinical Sample of Young People in Singapore: A Cross-Sectional Study

**DOI:** 10.3390/ijerph17041429

**Published:** 2020-02-23

**Authors:** Ellaisha Samari, Shazana Shahwan, Edimansyah Abdin, YunJue Zhang, Rajeswari Sambasivam, Wen Lin Teh, Say How Ong, Siow Ann Chong, Mythily Subramaniam

**Affiliations:** 1 Research Division, Institute of Mental Health, 10 Buangkok View, Buangkok Green, Medical Park, Singapore 539747, Singapore; shazana_mohamed_shahwan@imh.com.sg (S.S.); edimansyah_abdin@imh.com.sg (E.A.); yunjue_zhang@imh.com.sg (Y.Z.); rajeswari_sambasivam@imh.com.sg (R.S.); wen_lin_teh@imh.com.sg (W.L.T.); siow_ann_chong@imh.com.sg (S.A.C.); mythily@imh.com.sg (M.S.); 2 Department of Developmental Psychiatry, Institute of Mental Health, 10 Buangkok View, Buangkok Green, Medical Park, Singapore 539747, Singapore; say_how_ong@imh.com.sg

**Keywords:** deliberate self-harm, mental illness, young people, suicide attempt

## Abstract

This study examined differences between young people with mental illness who engage in deliberate self-harm with and without suicidal intent, as well as socio-demographic and clinical factors that are related to the increased likelihood of suicide attempt amongst self-harming young people. A total of 235 outpatients with mental illness who had engaged in deliberate self-harm were recruited from a tertiary psychiatric hospital in Singapore. Participants completed a self-report questionnaire which collected information on their socio-demographic background, self-harm history, diagnosis, depressive symptoms and childhood trauma. A total of 31.1% had reported a history of attempted suicide. Multiple logistic regression conducted found that engaging in self-harm ideation between 1 and 7 days (OR = 4.3, *p* = 0.30), and more than 1 week (OR = 10.5, *p* < 0.001) (versus no engagement in any self-harm ideation at all), were significantly associated with greater likelihood of attempted suicide. This study reports a relatively high prevalence rate of reported suicide attempts amongst young people with mental illness who engaged in self-harm. Identifying self-harm behaviors and treating it early could be the first step in managing potential suicidal behaviors among those who engage in self-harm.

## 1. Introduction

Self-injurious behaviors refer to a broad category of behaviors that result in direct and deliberate harm of one’s own body, that typically peaks during adolescent years [1,2]. Notably, a complex issue in this field of research is the use of various nomenclature in the literature with definitions differing based on the extent to which the behavior includes suicidal or non-suicidal intent. Consequently, comparing prevalence across studies and countries becomes challenging.

Nonetheless, existing literature suggests that a significant number of young people worldwide engage in self-injurious behaviors. Muehlenkamp et al. [3] conducted a systematic review across 52 studies of adolescent samples and found a mean lifetime prevalence of 18.0% for non-suicidal self-injury (NSSI) and 16.1% for deliberate self-harm (DSH). The high prevalence coupled with its strong associations with recurrence and suicidal behaviors make it a major health concern [3,4].

Terms such as “non-suicidal self-injury” (NSSI) and “deliberate self-harm” (DSH) are amongst the more commonly used terms in the literature. NSSI is used to denote the deliberate damage to one’s own body tissue without suicidal intent [5] while DSH is more frequently used as an encompassing term for self-injurious behavior both with and without suicidal intent that has non-fatal outcomes [4,6]. However, even across countries, similar terms are used to represent different acts. For example, while the term DSH is understood to encompass behaviors regardless of suicidal intent in publications originating from the UK [7], it is understood to exclude suicidal intent in the US. Nonetheless, given its more frequent use as an encompassing term which is in line with the investigation of our study, we will be using the term DSH in this paper to investigate self-injurious behaviors with and without suicidal intent amongst a sample of young people with mental illness. However, due to the varied terminologies used in the literature, references made from other research studies will use the terms that the authors of the respective studies have used. 

Various researchers have brought forth the notion that suicidal intent is difficult to operationalize and that there could be multiple reasons behind the behavior which may not always be clear and consistent [8]. In fact, many who had made an apparent suicide attempt had no intention to die and instead, used it as a way to temporarily escape a painful situation, cope with intolerable feelings, punish one’s self or as a cry for help [9,10]. Nonetheless, it is critical to note that making a suicide attempt can be a predictor of future suicide [11] as is a history of deliberate self-harm [12,13]. Studies have also shown that amongst those who had previously engaged in NSSI, 70% had attempted suicide at least once and 55% had multiple attempts [14,15]. 

Research has identified various factors that predispose adolescents to DSH including biological, psychological, social and cultural. Specifically, these risks include serotonin imbalances, exposure to negative life events, and psychiatric disorders. Childhood trauma resulting from sexual and physical abuse and maladaptive parenting is another factor associated with DSH during adolescence [16].

Non-suicidal and suicidal behaviors are generally distinct and have important clinical differences in terms of etiology, functions, methods, and course. For example, NSSI has been primarily associated with affect-regulation as its central function [17] and individuals who engage in such behaviors do so to alleviate negative emotions such as depression, anxiety and emotional distress which they cannot endure [17,18] to communicate with/influence others and as a form of self-punishment [19]. In contrast, suicidal behavior is primarily associated with the desire to die. However, previous research has found other motivations driving suicidal behavior amongst adolescents [10]. In a study done amongst females with a borderline personality disorder, researchers found that suicidal behaviors were more related to interpersonal reasons such as ‘to make others better off’ [20]. This idea resonates with Joiner’s [21] interpersonal theory of suicide who posit perceived burdensomeness and failed belongingness as driving factors for suicidal desire. Perceived burdensomeness represents the idea that one’s existence acts as a burden to his or her family, friends and/or society, thereby suggesting one’s death as more worthy than his or her life. Failed belongingness, on the other hand, is the experience of feeling alienated from others and not being an essential part of a family, friends or other desired groups. The theory argues that the combination of experiencing both perceived burdensomeness and failed belongingness simultaneously precipitates the notion that there this nothing left to live for, thereby resulting in the development of a desire for death [22].

The coexistence of NSSI and suicidal behavior has been frequently observed [15,16]. This urges us to try and understand the nature of their association and relationship. However, prior research has shown an equivocal nature of their relationship. Some have argued that NSSI is seen as a way to maintain life by regulating negative emotions [23] while others reason that NSSI is a factor that triggers the development of suicidal ideation and attempts [21].

Previous research done on NSSI amongst adolescents in Singapore examined the characteristics, functions and correlates of non-suicidal self-injury within a small clinical sample [24]. As for research on suicidal behavior, a cross-sectional epidemiological study conducted previously to examine socio-demographic and clinical correlates of suicidal behavior amongst Singapore residents aged 18 and above found the prevalence of suicide ideation, plan and attempt amongst those with lifetime major depressive disorder to be 43.6%, 13.7% and 12.3%, respectively [25]. 

Prior studies on deliberate self-harm were mainly conducted in Western countries such as the US and there is an underrepresentation of non-Western countries in the self-harm literature [26]. Importantly, various aspects of self-harm and suicidal behaviors are highly influenced by cultural contexts including gender differences, suicide rates, meaning of suicide and the methods used [27] For example, some researchers have noticed that suicide rates are highest amongst young people in Asian countries while it is highest for the elderly in Western countries and female to male ratio is higher in Asian countries when compared to Western countries [28,29]. Therefore, we sought to enrich the literature by analyzing these behaviors in a multi-ethnic Asian country—Singapore. Specifically, this study aims to identify differences between those who engage in self-harm with and without suicidal intent. Identification of such differences could enable better assessment and identification of self-harming young people who may be at risk for suicidal behavior.

## 2. Materials and Methods

Data from this study came from a larger cross-sectional study which examined deliberate self-harm amongst young people (14 to 35 years old) with mental illness in Singapore [30]. The age range of young people was derived based on two local definitions of young people: 1) The Children and Young Persons Act (1993) which defines a young person as 14–16 years old and 2) the National Youth Council which defines youth as those aged 15–35 years old [31]. While the original study involved a larger sample of participants, we only extracted data from participants who engaged in self-harm with or without suicidal intent for the purpose of our study. Participants were recruited from a tertiary care psychiatric hospital in Singapore through convenient sampling. All participants had provided informed consent prior to completing the questionnaires. Given the sensitive nature of the topic and thereby the possibility of adolescents concealing their self-harm behavior, a waiver of parental consent was applied to participants aged below 21 to minimize underreporting of such behaviors. Nonetheless, parents who accompanied participants to the hospital were briefed by researchers about the study. Participants’ clinical diagnoses were determined by a psychiatrist (using ICD-10 criteria). Ethical approval was obtained from the relevant institutional ethics review board (National Healthcare Group Domain Specific Review Board).

Participants were categorized into two groups on the basis of their self-harm history: self-harm without suicidal intent (DSH w/o SI) and self-harm with suicidal intent (DSH w/SI). Inclusion criteria for DSH w/o SI and DSH w/SI were history of any self-harm without and with suicidal intent in the past 12 months, respectively. 

### 2.1. Instruments

#### 2.1.1. Childhood Trauma Questionnaire-Short Form (CTQ-SF)

The Childhood Trauma Questionnaire-Short Form (CTQ-SF) [32] is a 28-item self-report instrument that was developed to measure the severity of childhood or adolescent abuse and neglect. The instrument consists of five subscales, three measuring abuse (emotional, physical, and sexual abuse) and two measuring neglect (emotional and physical neglect). Each item is measured on a 5-point Likert scale ranging from never true (1) to very often true (5). Items in this scale are summed to yield a total score. Higher scores indicate a more severe experience of childhood or adolescent maltreatment. The CTQ-SF has also been shown to have strong reliability and validity [32,33] in prior research. Cronbach’s alpha in the present study was 0.71.

#### 2.1.2. Patient Health Questionnaire (PHQ-8)

Depressive symptoms were measured using the eight-item Patient Health Questionnaire (PHQ-8); a self-report questionnaire that was designed to measure the severity of depressive symptoms [34]. Each item is valued from 0 (not at all) to 3 (nearly every day). Items in this scale are summed to yield a total score. Higher scores on the scale would indicate greater severity of depressive symptoms. The reliability and validity of the PHQ-8 has been supported in prior research [33]. Cronbach’s alpha in the present study was 0.89.

#### 2.1.3. Functional Assessment of Self-Mutilation (FASM)

The Functional Assessment of Self-Mutilation (FASM) [35] was used to assess methods, frequency and functions of self-reported self-harm in the past year. Participants were asked if they had engaged in any of 11 listed self-harm behaviors in the past 12 months, the number of times they engaged in each behavior, the length of time they took to contemplate the behavior (self-harm ideation) and whether any of these behaviors was a suicide attempt. A single question was used to divide the present sample into two groups (DSH w/o SI and DSH w/SI)—“While doing any of the above acts (11 listed self-harm behaviors), were you trying to kill yourself?”. Participants who answered “no” formed the DSH w/o SI group while participants who answered “yes” formed the DSH w/SI group. 

To assess the reasons for self-harm, participants were given a list of 22 reasons for engaging in self-harm to which they were asked to respond with either “Never (0)”, “Rarely (1)”, “Some (2)” or “Often (3)”. The reliability and validity of the FASM has been supported in prior research [5,36].

### 2.2. Statistical Analysis

All statistical analyses in the present study were performed using the IBM SPSS statistics version 23. Descriptive statistics were calculated for the overall sample and the two groups (DSH w/o SI and DSH w/SI). Frequencies and percentages were calculated for categorical variables and mean and standard deviations were calculated for continuous variables. 

Chi-square test and t-tests were conducted to determine whether there were any significant differences in the categorical (gender, ethnicity, diagnosis, education, number of different self-harm methods engaged in, and length of self-injury ideation) and continuous (age, PHQ-8 score, and CTQ subscales scores) variables respectively between the two groups (DSH w/o SI and DSH w/SI). Mann–Whitney U test was also conducted to compare ordinal variables (endorsement of reasons for self-harm) between the two groups (DSH w/o SI and DSH w/SI).

Multiple logistic regression was done to examine the associations between sociodemographic and clinical variables and attempted suicide. To prevent overloading of the model due to large number of variables with small sample size, we carried out the following steps: (1) A series of simple logistic regressions were done for each of the aforementioned variables (2) Variables that were significantly associated with attempted suicide (*p* < 0.05) were added into the initial multiple logistic regression model using enter method (3) Following that, we eliminated non-significant variables (*p* > 0.05) and conducted the multiple logistic regression analysis again to identify the final model. 

Listwise deletion was used to handle missing data and all statistically significant results are reported at *p* < 0.05.

## 3. Results

Table 1 shows the socio-demographic information, duration of self-harm ideation, number of different methods of self-harm engaged in, primary diagnosis of patients as well as mean PHQ-8 and CTQ-SF subscales scores across the two groups (DSH w/o SI and DSH w/SI). The present sample population consisted of 235 participants with a mean age of 21.9 years old (SD = 5.6; range 14 to 35 years). A total of 58.3% were female, 68.1% were Chinese and 42.6% were either in the process of completing or had completed post-secondary education. A total of 43.8% had a primary diagnosis of mood disorders. Overall, of the 235 patients who had engaged in self-harm behavior, 31.1% reported a history of attempted suicide.

### 3.1. Methods of Self-Harm

As seen in Table 1, most of the participants in the DSH w/o SI group had engaged in one method of self-harm only (30.6%) while most of the participants in the DSH w/SI group had engaged in five or more methods of self-harm (41.1%). 

Table 2 presents the different methods of self-harm by the two groups (DSH w/o SI and DSH w/SI). In both groups, “hit self on purpose” (DSH w/o SI—59.9%; DSH w/SI—82.2%) was the most common method of self-harm that participants had engaged in. For the DSH w/o SI group, the next common method of self-harm engaged in was “cut or carved on skin” (41.4%) followed by “picked at wound” (32.7%) and “bit self” (32.7%). For the DSH w/SI group, the next common method of self-harm engaged in was also “cut or carved on skin” (75.3%) followed by “bit self” (49.3%). 

### 3.2. Functions of Self-Harm

Table 3 displays the reasons for self-harm by two self-harm groups (DSH w/o SI and DSH w/SI). In both groups, “to relieve feeling “numb” or empty”, “to punish yourself”, and “to stop bad feelings” were the top three reasons for engaging in self-harm based on their median scores. The frequency of engaging in self-harm for all three reasons were also significantly higher for participants in the DSH w/SI group as compared to participants in the DSH w/o SI group: “to relieve feeling “numb” or empty”—DSH w/SI group: Mdn = 3.00; DSH w/o SI group: Mdn = 2.00, U=3958.5, *p* < 0.001); “to punish yourself”—DSH w/SI group: Mdn = 3.00; DSH w/o SI group: Mdn = 2.00, U = 3421.0, *p* < 0.001); “to stop bad feelings”—DSH w/SI group: Mdn = 3.00; DSH w/o SI group: Mdn = 2.00, U = 4155.0, *p* < 0.001).

### 3.3. Correlates of History of Suicide Attempt

As seen from Table 4, higher PHQ scores (OR = 1.1, *p* = 0.001) were significantly associated with higher likelihood of attempted suicide. Engaging in self-harm ideation between 1 and 7 days (OR = 4.3, *p* = 0.30), and greater than 1 week (OR = 10.5, *p* < 0.001) were significantly associated with higher likelihood of attempted suicide as compared to no engagement in any self-harm ideation at all. Engaging in 5 or more methods of self-harm (OR = 5.8, *p* = 0.001) was also significantly associated with higher likelihood of attempted suicide as compared to engaging in 1 method of self-harm. 

## 4. Discussion

The present study explored socio-demographic and clinical differences between a clinical sample of young people who had engaged in deliberate self-harm without suicidal intent (DSH w/o SI) and those who had engaged in deliberate self-harm with suicidal intent (DSH w/SI). Overall, results from this study showed several similarities and differences that hold important clinical implications. 

Results from our study highlight some interesting findings concerning methods of self-harm employed by both groups (DSH w/o SI and DSH w/SI). Both groups reported ‘hit self on purpose’ and ‘cut or carved on skin’ as the two most common methods of self-harm with a higher percentage of participants in the DSH w/SI group (82.2%; 75.3%) engaging in these behaviors as compared to participants in the DSH w/o SI group (59.9%; 41.4%). Cutting and carving on one’s skin can be considered as one of the more lethal methods of self-harm. According to prior studies, there are some methods and characteristics of self-harm which appear to be at higher risk of leading to suicidal behavior than others and cutting is one of them [15,37]. Furthermore, the lethality of methods has been identified as one of the distinguishing factors between those who engaged in non-suicidal self-harm alone and those who attempted suicide [21].

However, we are unable to determine which act specifically that participants had engaged in were with or without suicidal intent. Nevertheless, similar patterns of self-harm behaviors reported by these two groups seem to question the act’s communicative value and what the desire to die can mean to young people. Therefore, future research measuring the severity and consequence of the methods of self-harm which they used when attempting suicide would be useful in understanding the value of these acts and the construct of death among this group of people.

Notably, engaging in five or more methods of self-harm was associated with a higher likelihood of having attempted suicide as compared to engaging in one method of self-harm only. Prior research found a positive association between the number of NSSI methods and a history of suicide attempts. It was suggested that engaging in numerous amounts of NSSI methods may have led to varied painful and stimulating experiences which contributed to the habituation of fear and pain associated with these acts [21,38]. Furthermore, Joiner [21] also theorized that the habituation of the act itself and its associated pain may result in attaining greater capability to perform more lethal forms of self-harm. However, given the nature of our study, we are unable to ascertain if self-harm without suicide intent was preceded by those with suicide intent thus rendering any firm interpretations difficult. 

Interestingly, the top three reasons for self-harm were similar for both the DSH w/o SI and DSH w/SI group which include “to relieve feeling “numb” or empty”, “to punish yourself” and “to stop bad feelings”. Previously, Nock and Prinstein [5,36] had developed a four-factor model of NSSI functions from the FASM. The four functions include: automatic negative reinforcement (ANR), automatic positive reinforcement (APR), social negative reinforcement (SNR) and social positive reinforcement (SPR). Based on this model, “to relieve feeling “numb” or empty” and “to stop bad feelings” both serve the ANR function of reducing bad feelings. This resonates with previous research which mainly supports an affect regulating function of NSSI [36]. In fact, similar results were obtained in previous studies done in a community sample in Singapore [24] and Sweden [39]. On the other hand, “to punish self” serves the SNR function and this supports the findings of previous research which found that self-directed anger, shame, and hatred predicted NSSI acts [40].

We found that taking a longer time to contemplate self-harm was more likely to be associated with having attempted suicide. Various possibilities could account for this association. One of which is impulsivity. Our results suggest that participants belonging to the DSH w/SI group may be less impulsive as compared to participants in the DSH w/o SI group. On the contrary, a previous study exploring impulsivity traits amongst psychiatric adolescent patients who engaged in NSSI with and without suicide attempt in the US found that those who had attempted suicide exhibited greater signs of impulsivity on standard clinical measures [41]. Similarly, Mexican psychiatric patients with a history of suicide attempt also had higher impulsivity traits compared to those who engaged in NSSI only [42]. However, it is important to note that the measures of impulsivity in these studies were not directly related to engagement of self-harm, unlike the question in our study. Recent literature has also suggested that repetitive self-harm could be considered as an addictive behavior [43]. Based on this notion, it can be suggested that these individuals are dealing with strong urges to harm themselves. It is therefore possible that variation in time taken to contemplate before engaging in the act shows differences in level of resistance against these urges. Understanding the underlying reasons and nature of self-harm for each individual is crucial in tailoring a more suitable and effective treatment. 

As expected, higher PHQ-8 scores (more severe depressive symptoms) were significantly associated with an increased likelihood of having attempted suicide. In fact, one of the strongest risk factors found previously for completed and attempted suicide in adolescents is a psychiatric diagnosis [44] and commonly, depressive disorder [45]. As mentioned in the literature, individuals engage in self-harm for various reasons, including the desire to alleviate negative emotions such as depression, anxiety, and emotional distress which they cannot endure [17,18]. Therefore, there is a chance that those with more severe depressive symptoms were more overwhelmed by such negative emotions and see death as the only way out, and thus attempted suicide. However, it is important to note that while depressive symptoms increase risk of suicide, the majority of young people with depression do not attempt suicide. Hence it is vital that we continue investigating other important risk factors for suicidal behavior amongst young people with depressive symptoms in order to tailor better prevention and treatment efforts for them. 

The present study has various strengths including a relatively large sample size of clinical population and the use of standardized questionnaires. Furthermore, it is one of the few studies in Asia that looks into comparing differences between those who engage in self-harm with and without suicidal intent. However, the present study holds a few limitations. There is an unequal representation of the two groups (DSH w/o SI and DSH w/SI) hence limiting generalizability of results. Future studies that attempt to expand on these findings should aim to increase sample sizes for the DSH w/SI group. Additionally, as this study is cross-sectional in nature, it precludes the discovery of any causal relationships. Therefore, prospective studies examining self-harm in young people are greatly needed. As convenience sampling was used for the present study, the sample is not representative of the entire population and therefore limits the generalizability of this study’s findings. Although it is a self-report measure and anonymity of data has been ensured, responses may be affected by social desirability bias given the sensitive nature of the topic. In terms of analysis, lower number of events per covariate are needed to obtain reliable estimates of regression coefficients when fitting a logistic regression model. Peduzzi et al. [46] recommended that a minimum of 10 events per parameter are needed to avoid problems of overestimation or underestimation of the sample variance of the regression coefficients. In our study, out of 235, 73 subjects reported having DSH w/SI. If we follow the criteria which require a minimum of 10 events per parameter, our regression model should have no more than 7 parameters in one single model. Hence, exceeding 7 parameters in our regression model may affect the reliability of our regression estimates. Hence, to overcome this problem, we used a stepwise regression approach where only significant predictors from simple logistic regression analyses were included in the final model.

## 5. Conclusions

Overall, this study reports a relatively high prevalence rate of reported suicide attempts amongst young people with mental illness who engaged in self-harm and highlights the differences between those who engaged in self-harm with and without suicidal intent. While previous studies have found various differences, the present study included a number of variables into the regression model which has been scarcely researched on in Asia. Awareness of risk factors such as duration of self-harm ideation and depressive symptoms could be beneficial for treatment of young people who are at risk for suicidal behaviors. Similar engagement of self-harm behaviors between both groups also suggests that similar behaviors can be done with or without the intention to die.

It is also crucial to manage potential suicidal behaviors among those who engage in self-harm. Identifying self-harm behaviors and treating it early could also be the first step in preventing the development of suicidal behavior during their life course. Additionally, results also suggest further examination about the communicative value of the act and its functions. Addressing these concerns during treatment could prevent maladaptive coping strategies to deal with the distress they are facing. Furthermore, self-harm is quite a common behavior amongst young people and therefore identifying specific factors that may increase risk of suicidal behaviors is also very important, not just at the clinical level, but at all levels from teachers and peers in school to friends and family at home. Lastly, it is imperative that we do not underestimate the gravity of self-harm and its association with suicidal behaviors. 

## Figures and Tables

**Table 1:** Socio-demographic and clinical profile of study sample.

Socio-Demographic and Clinical Variables		Overall		DSH w/o SI		DSH w/SI		*p* Value
		*n*	%	*n*	%	*n*	%	
		235		162	68.9	73	31.1	
Gender	Female	137	58.3	88	54.3	49	67.1	0.065
	Male	98	41.7	74	45.7	24	32.9	
Ethnicity	Chinese	160	68.1	118	72.8	42	57.5	0.064
	Malay	42	17.9	22	13.6	20	27.4	
	Indian	23	9.8	15	9.3	8	11.0	
	Others (Specify)	10	4.3	7	4.3	3	4.1	
Diagnosis	Adjustment disorders	48	20.4	32	19.8	16	21.9	0.143
	Anxiety disorders	26	11.1	19	11.7	7	9.6	
	Childhood disorders	9	3.8	9	5.6	0	0.0	
	Mood disorders	103	43.8	65	40.1	38	52.1	
	Schizophrenia and psychotic disorders	34	14.5	24	14.8	10	13.7	
	Others	15	6.4	13	8.0	2	2.7	
Highest education	Primary	22	9.4	12	7.4	10	13.7	0.151
	Secondary	99	42.1	64	39.5	35	47.9	
	Post-Secondary	100	42.6	75	46.3	25	34.2	
	Degree and above	14	6.0	11	6.8	3	4.1	
Self-harm ideation	None	81	34.5	66	40.7	15	20.5	<0.001
	A few minutes	81	34.5	60	37.0	21	28.8	
	Less than 60 min	25	10.6	16	9.9	9	12.3	
	Between 1 to 24 hours	12	5.1	7	4.3	5	6.8	
	Between 1 to 7 days	14	6.0	7	4.3	7	9.6	
	Greater than a week	22	9.4	6	3.7	16	21.9	
Number of self-harm methods engaged in	1	72	30.6	62	38.3	10	13.7	<0.001
	2	59	25.1	44	27.2	15	20.5	
	3 to 4	57	24.3	39	24.1	18	24.7	
	5 and above	47	20.0	17	10.5	30	41.1	
		**M**	**SD**	**M**	**SD**	**M**	**SD**	***p* value**
Age		21.9	5.6	21.8	5.3	22.2	6.3	0.023
PHQ-8 score	Depressive symptoms	13.0	6.6	11.5	6	16.5	6	<0.001
CTQ-28 score	Emotional abuse	13.6	5.7	12.5	5	16.4	6	<0.001
	Physical abuse	10.1	5.4	9.3	5	12.0	6	0.001
	Sexual abuse	7.5	5.0	7.2	4	8.2	6	0.185
	Emotional neglect	14.4	5.7	13.7	5	16.3	6	0.002
	Physical neglect	8.9	3.3	8.4	3	10.0	4	0.001

**Table 2:** Frequency and percentage of self-reported deliberate self-harm (DSH) in the past 12 months.

**DSH w/o SI**	**Ever Engaged in Behavior in Past 12 Months**		**No. of Times in Past 12 Months**							
			**Once**		**2 to 5**		**6 to 10**		**>10**	
**Method of DSH**	***n***	**%**	***n***	**%**	***n***	**%**	***n***	**%**	***n***	**%**
Cut or carved on skin	67	41.4	12	17.9	37	55.2	8	11.9	10	14.9
Hit self on purpose	97	59.9	9	9.3	47	48.5	20	20.6	19	19.6
Pulled hair out	28	17.3	7	25.0	11	39.3	3	10.7	7	25.0
Gave self a tattoo	16	9.9	4	25.0	9	56.3	0	0.0	1	6.3
Picked at a wound	53	32.7	6	11.3	33	62.3	7	13.2	6	11.3
Burned skin	15	9.3	0	0.0	6	40.0	0	0.0	1	6.7
Inserted objects under nails or skin	4	2.5	0	0.0	4	100.0	0	0.0	0	0.0
Bit self	53	32.7	8	15.1	19	35.8	9	17.0	17	32.1
Picked areas of body to the point of drawing blood	18	11.1	2	11.1	8	44.4	2	11.1	6	33.3
Scraped skin	27	16.7	3	11.1	18	66.7	2	7.4	4	14.8
Erased skin	4	2.5	1	25.0	3	75.0	0	0.0	0	0.0
**DSH w/SI**			**Once**		**2 to 5**		**6 to 10**		**>10**	
**Method of DSH**	***n***	**%**	***n***	**%**	***n***	**%**	***n***	**%**	***n***	**%**
Cut or carved on skin	55	75.3	4	7.3	24	43.6	11	20	15	27.3
Hit self on purpose	60	82.2	4	6.7	20	33.3	13	21.7	23	38.3
Pulled hair out	32	43.8	1	3.1	14	43.8	4	12.5	13	40.6
Gave self a tattoo	8	11	3	37.5	2	25	2	25	1	12.5
Picked at a wound	30	41.1	2	6.7	11	36.7	6	20	11	36.7
Burned skin	14	19.2	2	14.3	4	28.6	1	7.1	3	21.4
Inserted objects under nails or skin	9	12.3	1	11.1	3	33.3	1	11.1	4	44.4
Bit self	36	49.3	2	5.6	13	36.1	6	16.7	15	41.7
Picked areas of body to the point of drawing blood	25	34.2	1	4	9	36	7	28	8	32
Scraped skin	24	32.9	2	8.3	11	45.8	1	4.2	10	41.7
Erased skin	7	9.6	0	0	2	28.6	2	28.6	3	42.9

**Table 3:** Endorsement of reasons for self-harm.

Reasons for Self-Harm	Median		Mann-Whitney U	*p* Value
	DSH w/o SI	DSH w/SI		
1. to avoid school, work or other activities	0.00	1.00	4288.500	<0.001
2. to relieve feeling “numb” or empty	2.00	3.00	3958.500	<0.001
3. to get attention	0.00	1.00	4830.500	0.014
4. to feel something, even if it was pain	1.00	2.00	3647.500	<0.001
5. to avoid having to do something unpleasant you do not want to do	0.00	1.00	3915.500	<0.001
6. to get control of a situation	1.00	2.00	4504.500	0.002
7. to try to get a reaction from someone, even if it is a negative reaction	0.00	1.00	4189.000	<0.001
8. to receive more attention from your parents or friends	0.00	1.00	4185.500	<0.001
9. to avoid being with people	0.00	1.00	4256.000	<0.001
10. to punish yourself	2.00	3.00	3421.000	<0.001
11. to get other people to act differently or change	0.00	1.00	4423.000	<0.001
12. to be like someone you respect	0.00	0.00	5431.500	0.162
13. to avoid punishment or paying the consequences	0.00	0.00	4971.500	0.015
14. to stop bad feelings	2.00	3.00	4155.000	<0.001
15. to let others know how desperate you were	0.00	1.00	4676.000	0.003
16. to feel more a part of a group	0.00	0.00	5469.000	0.187
17. to get your parents to understand or notice you	0.00	1.00	4185.000	<0.001
18. to give yourself something to do when alone	0.00	1.00	4736.500	0.006
19. to give yourself something to do when with others	0.00	0.00	5461.000	0.193
20. to get help	0.00	0.00	4345.500	<0.001
21. to make others angry	0.00	0.00	5141.500	0.026
22. to feel relaxed	1.00	2.00	4016.000	<0.001

**Table 4:** Sociodemographic and clinical variables associated with attempted suicide.

Sociodemographic and Clinical Variables	Crude OR ^†^	95% C.I.		*p* Value ^‡^	Adjusted OR ^§^	95% C.I.		*p* Value
**Age**	1.01	0.96	1.06	0.645				
**Gender**								
Female	0.58	0.33	1.04	0.067				
Male	Ref							
**Ethnic group**								
Malay	2.55	1.27	5.15	0.009				
Indian	1.50	0.59	3.79	0.393				
Others	1.20	0.30	4.87	0.795				
Chinese	Ref							
**Diagnosis**								
Adjustment disorders	0.86	0.42	1.76	0.671				
Anxiety disorders	0.63	0.24	1.64	0.343				
Childhood disorders	.	.	.	.				
Schizophrenia and psychotic disorders	0.71	0.31	1.65	0.429				
Others	0.26	0.06	1.23	0.090				
Mood disorders	Ref							
**Education level**								
Secondary	0.66	0.26	1.67	0.377				
Post-secondary	0.40	0.15	1.04	0.060				
Degree and above	0.33	0.07	1.51	0.152				
Primary	Ref							
**Duration of self-harm ideation**								
A few minutes	1.54	0.73	3.26	0.259	1.61	0.69	3.71	0.268
Less than 60 min	2.48	0.92	6.67	0.073	2.57	0.85	7.80	0.096
Between 1 and 24 hours	3.14	0.88	11.27	0.079	1.50	0.37	6.02	0.567
Between 1 and 7 days	4.40	1.34	14.44	0.015	4.31	1.15	16.15	0.030
Greater than a week	11.7	3.93	35.00	<0.001	10.48	3.19	34.43	<0.001
None	Ref							
**Number of self-harm methods**								
2	2.11	0.87	5.14	0.099	1.224	0.45	3.31	0.690
3 to 4	2.86	1.20	6.84	0.018	1.696	0.64	4.48	0.286
5 and above	10.9	4.47	26.76	<0.001	5.764	2.07	16.06	0.001
1	Ref							
**PHQ-8 score**	1.14	1.09	1.20	<0.001	1.10	1.04	1.17	0.001
**CTQ-SF score**								
Emotional abuse	1.13	1.07	1.20	<0.001				
Physical abuse	1.10	1.04	1.16	0.001				
Sexual abuse	1.04	0.98	1.10	0.188				
Emotional neglect	1.09	1.03	1.15	0.003				
Physical neglect	1.16	1.06	1.26	0.001				

Socio-demographics factors such as age, gender, ethnic group, diagnosis, education level as well as clinical factors such as PHQ-8 score and CTQ-SF subscale scores were included in the logistic regression. ^†^ Simple Logistic Regression. ^‡^ Likelihood-ratio test, *p*-value <0.05. § Multiple logistic regression. Ref—Reference group; CI—confidence Interval; OR—odd ratio; PHQ—Patient Health Questionnaire; CTQ-SF—Childhood Trauma Questionnaire-Short Form.

## References

[1] Nock M.K. (2010). Self-Injury. Annu. Rev. Clin. Psychol..

[2] Whitlock J. (2010). Self-Injurious behavior in adolescents. PLoS Med..

[3] Hawton K., Bergen H., Casey D., Simkin S., Palmer B., Cooper J., Kapur N., Horrocks J., House A., Lilley R. (2007). Self-Harm in England: A tale of three cities. Soc. Psychiatry Psychiatr. Epidemiol..

[4] Muehlenkamp J.J., Claes L., Havertape L., Plener P.L. (2012). International prevalence of adolescent non-Suicidal self-Injury and deliberate self-Harm. Child Adolesc. Psychiatry Ment. Health.

[5] Nock M.K., Prinstein M.J. (2005). Contextual features and behavioral functions of self-Mutilation among adolescents. J. Abnorm. Psychol..

[6] Madge N., Hewitt A., Hawton K., Wilde E.J., Corcoran P., Fekete S., Heeringen K.V., Leo D.D., Ystgaard M. (2008). Deliberate self-Harm within an international community sample of young people: Comparative findings from the Child & Adolescent Self-harm in Europe (CASE) Study. J. Child Psychol. Psychiatry.

[7] Fliege H., Lee J.R., Grimm A., Klapp B.F. (2009). Risk factors and correlates of deliberate self-Harm behavior: A systematic review. J. Psychosom. Res..

[8] Kapur N., Cooper J., O’Connor R.C., Hawton K. (2013). Non-Suicidal self-injury v. attempted suicide: New diagnosis or false dichotomy?. Br. J. Psychiatry.

[9] Hjelmeland H., Knizek B.L., Nordvik H. (2002). The communicative aspect of nonfatal suicidal behavior: Are there gender differences?. Crisis.

[10] Rodham K., Hawton K., Evans E. (2004). Reasons for deliberate self-Harm: Comparison of self-Poisoners and self-Cutters in a community sample of adolescents. J. Am. Acad. Child Adolesc. Psychiatry.

[11] Suominen K.M., Henriksson M., Suokas J., Isometsä E., Ostamo A., Lönnqvist J. (1996). Mental disorders and comorbidity in attempted suicide. Acta Psychiatr. Scand..

[12] Cooper J., Kapur N., Webb R., Lawlor M., Guthrie E., Mackway-Jones K., Appleby L. (2005). Suicide after deliberate self-harm: A 4-Year cohort study. Am. J. Psychiatry.

[13] Klonsky E.D., Victor S.E., Saffer B.Y. (2014). Nonsuicidal self-Injury: What we know, and what we need to know. Can. J. Psychiatry.

[14] Hargus E., Hawton K., Rodham K. (2009). Distinguishing between subgroups of adolescents who self-Harm. Suicide Life Threat Behav..

[15] Nock M.K., Joiner Jr T.E., Gordon K.H., Lloyd-Richardson E., Prinstein M.J. (2006). Non-Suicidal self-Injury among adolescents: Diagnostic correlates and relation to suicide attempts. Psychiatry Res..

[16] Hawton K., Saunders K.E., O’Connor R.C. (2012). Self-Harm and suicide in adolescents. Lancet.

[17] Fox K.R., Franklin J.C., Ribeiro J.D., Kleiman E.M., Bentley K.H., Nock M.K. (2015). Meta-Analysis of risk factors for nonsuicidal self-Injury. Clin. Psychol. Rev..

[18] Ross S., Heath N. (2002). A study of the frequency of self-Mutilation in a community sample of adolescents. J. Youth Adolesc..

[19] Swannell S., Martin G., Scott J., Gibbons M., Gifford S. (2008). Motivations for self-Injury in an adolescent inpatient population: Development of a self-Report measure. Australas. Psychiatry.

[20] Brown M.Z., Comtois K.A., Linehan M.M. (2002). Reasons for suicide attempts and nonsuicidal self-Injury in women with borderline personality disorder. J. Abnorm. Psychol..

[21] Joiner T. (2007). Why People Die by Suicide.

[22] Joiner  T.E., Van Orden  K.A. (2008). The interpersonal–Psychological theory of suicidal behavior indicates specific and crucial psychotherapeutic targets. Int. J. Cogn. Ther..

[23] Walsh B.W. (2012). Treating Self-Injury: A Practical Guide.

[24] Tan A.C., Rehfuss M.C., Suarez E.C., Parks-Savage A. (2014). Nonsuicidal self-Injury in an adolescent population in Singapore. Clin. Child Psychol. Psychiatry.

[25] Subramaniam M., Abdin E., Seow E.L., Picco L., Vaingankar J.A., Chong S.A. (2017). Suicidal ideation, suicidal plan and suicidal attempts among those with major depressive disorder. Ann. Acad. Med. Singap..

[26] Hamza C.A., Stewart S.L., Willoughby T. (2012). Examining the link between nonsuicidal self-Injury and suicidal behavior: A review of the literature and an integrated model. Clin. Psychol. Rev..

[27] Colucci E., Lester D. (2012). Suicide and Culture: Understanding the Context.

[28] Mayer P., Ziaian T. (2002). Suicide, gender, and age variations in India: Are women in Indian society protected from suicide?. Crisis.

[29] Vijayakumar L. (2005). Suicide and mental disorders in Asia. Int. Rev. Psychiatry.

[30] Shahwan S., Abdin E., Zhang Y., Sambasivam R., Fauziana R., Mahesh M., Ong S.H., Chong S.A., Subramaniam M. (2018). Deliberate Self-Harm in Psychiatric Outpatients Aged 14–35 Years in Singapore. Ann. Acad. Med. Singap..

[31] Youthpolicy.org Definition of Youth. http://www.youthpolicy.org/factsheets/country/singapore/.

[32] Bernstein D.P., Stein J.A., Newcomb M.D., Walker E., Pogge D., Ahluvalia T., Stokes J., Handelsman L., Medrano M., Desmond D. (2003). Development and validation of a brief screening version of the Childhood Trauma Questionnaire. Child Abus. Negl..

[33] Spinhoven P., Penninx B.W., Hickendorff M., van Hemert A.M., Bernstein D.P., Elzinga B.M. (2014). Childhood Trauma Questionnaire: Factor structure, measurement invariance, and validity across emotional disorders. Psychol. Assess..

[34] Kroenke K., Strine T.W., Spitzer R.L., Williams J.B., Berry J.T., Mokdad A.H. (2009). The PHQ-8 as a measure of current depression in the general population. J. Affect. Disord..

[35] Lloyd E.E., Kelley M.L., Hope T. Self-Mutilation in a community sample of adolescents: Descriptive characteristics and provisional prevalence rates. Proceedings of the Annual Meeting of the Society for Behavioral Medicine.

[36] Nock M.K., Prinstein M.J. (2004). A functional approach to the assessment of self-Mutilative behavior. J. Consult Clin. Psychol..

[37] Favazza A.R. (1998). The coming of age of self-Mutilation. J. Nerv. Ment. Dis..

[38] Victor S.E., Klonsky E.D. (2014). Correlates of suicide attempts among self-injurers: A meta-Analysis. Clin. Psychol. Rev..

[39] Zetterqvist M., Lundh L.G., Dahlström Ö., Svedin C.G. (2013). Prevalence and function of non-Suicidal self-Injury (NSSI) in a community sample of adolescents, using suggested DSM-5 criteria for a potential NSSI disorder. J. Abnorm. Child Psychol..

[40] Nock M.K., Prinstein M.J., Sterba S.K. (2009). Revealing the form and function of self-Injurious thoughts and behaviors: A real-Time ecological assessment study among adolescents and young adults. J. Abnorm. Psychol..

[41] Dougherty D.M., Mathias C.W., Marsh-Richard D.M., Prevette K.N., Dawes M.A., Hatzis E.S., Palmes G., Nouvion S.O. (2009). Impulsivity and clinical symptoms among adolescents with non-Suicidal self-Injury with or without attempted suicide. Psychiatry Res..

[42] Fresán A., Camarena B., González-Castro T.B., Tovilla-Zárate C.A., Juárez-Rojop I.E., López-Narváez L., González-Ramón A.E., Hernández-Díaz Y. (2016). Risk-Factor differences for nonsuicidal self-Injury and suicide attempts in Mexican psychiatric patients. Neuropsychiatr. Dis. Treat..

[43] Blasco-Fontecilla H., Fernández-Fernández R., Colino L., Fajardo L., Perteguer-Barrio R., de Leon J. (2016). The addictive model of self-Harming (non-Suicidal and suicidal) behavior. Front. Psychiatry.

[44] Beautrais A.L. (2001). Child and young adolescent suicide in New Zealand. Aust. N. Z. J. Psychiatry.

[45] Foley D.L., Goldston D.B., Costello E.J., Angold A. (2006). Proximal psychiatric risk factors for suicidality in youth: The Great Smoky Mountains Study. Arch. Gen. Psychiatry.

[46] Peduzzi P., Concato J., Kemper E., Holford T., Feinstein A. (1996). A simulation study of the number of events per variable in logistic regression analysis. J. Clin. Epidemiol..

